# Validation of the Short-Form Health Literacy Questionnaire (HLS-SF12) and Its Determinants among People Living in Rural Areas in Vietnam

**DOI:** 10.3390/ijerph16183346

**Published:** 2019-09-11

**Authors:** Tuyen Van Duong, Thao T. P. Nguyen, Khue M. Pham, Kien T. Nguyen, Manh H. Giap, Tung D. X. Tran, Chi X. Nguyen, Shwu-Huey Yang, Chien-Tien Su

**Affiliations:** 1 School of Nutrition and Health Sciences, Taipei Medical University, Taipei 110-31, Taiwan; duongtuyenvna@gmail.com (T.V.D.); sherry@tmu.edu.tw (S.-H.Y.); 2 Health Management Training Institute, Hue University of Medicine and Pharmacy, Thua Thien Hue 491-20, Vietnam; ntpthao.hmti@huemed-univ.edu.vn; 3 Faculty of Public Health, Hai Phong University of Medicine and Pharmacy, Hai Phong 042-12, Vietnam; pmkhue@hpmu.edu.vn; 4 Department of Health Education, Faculty of Social Sciences, Behavior and Health Education, Hanoi University of Public Health, Hanoi 119-10, Vietnam; ntk1@huph.edu.vn; 5 Emergency Department, Bai Chay Hospital, Quang Ninh 011-21, Vietnam; ghmanh@gmail.com; 6 School of Dentistry, Taipei Medical University, Taipei 110-31, Taiwan; d204105004@tmu.edu.tw; 7 Stem Cell Unit, Van Hanh Hospital, Ho Chi Minh City 725-10, Vietnam; 8 Department of Training and Direction of Healthcare Activities, Thu Duc District Hospital, Ho Chi Minh City 713-11, Vietnam; xuanchi8485@gmail.com; 9 Research Center of Geriatric Nutrition, Taipei Medical University, Taipei 110-31, Taiwan; 10 Nutrition Research Center, Taipei Medical University Hospital, Taipei 110-31, Taiwan; 11 School of Public Health, Taipei Medical University, Taipei 110-31, Taiwan; 12 Department of Family Medicine, Taipei Medical University Hospital, Taipei 110-31, Taiwan

**Keywords:** health literacy, HLS-SF12, validation, determinant, mountaineer, rural areas, education, health-related TV, ability to pay for medication, Vietnam

## Abstract

*Background:* Health literacy (HL) is an important factor in improving health inequalities in poor and marginalized groups. Assessing comprehensive HL is critical. In this study, we validated the use of a comprehensive short-form HL survey tool (HLS-SF12) and examined the determinants of HL among people in rural areas. *Methods:* A cross-sectional study was conducted in July 2019 on 440 people residing in mountainous areas in Vietnam. Health literacy was measured using the HLS-SF12. Personal characteristics were also collected. We analyzed data using confirmatory factor analysis, internal consistency analysis, and regression analysis. *Results:* The questionnaire demonstrated a good construct validity with satisfactory goodness-of-fit indices and item-scale convergent validity. The tool was reliable and homogeneous with Cronbach’s alpha = 0.79, with no floor/ceiling effects. People who were married had lower HL (regression coefficient B = −3.12; 95% confidence interval (CI) = −5.69, −0.56; *p* = 0.017) compared with those who never married. Higher education attainment (B = 3.41 to 10.44; *p* < 0.001), a better ability to pay for medication (B = 4.17 to 9.89; *p* < 0.001), and a tendency to view health-related TV/radio more often (B = 5.23 to 6.15; *p* < 0.001) were associated with higher HL. *Conclusions:* The HLS-SF12 is a valid survey tool for the evaluation of HL in rural populations. A number of personal characteristics were strongly associated with HL.

## 1. Introduction

The commitment of the 2030 Sustainable Development Goals (SDGs) agenda is that “no one will be left behind” [1]. Education and literacy are set as one of the main goals of the plan [1]. Literacy and schooling can empower people’s awareness and improve critical thinking skills about their sustainable health, especially in vulnerable groups [1,2]. In addition, health literacy is also considered to be an integral part of policy domains aimed at improving health inequalities within the context of SDGs [3]. Health literacy (HL) has been comprehensively defined as “the knowledge, motivation, and competence to access, understand, appraise, and apply information in everyday life to make judgments and decisions about health care, disease prevention, and health promotion, and to maintain and promote quality of life throughout the life course” [4].

Non-communicable diseases (NCDs) are a heavy burden to Vietnam and the world [5,6]. The strategical interventions to prevent and manage NCD and its burden is essential to advance SDGs, especially in low- and middle-income countries [7,8]. The trend of disease in Vietnam shifted from communicable to non-communicable during the socio-economic reforms of the country from a poor to a low- to middle-income country [9,10,11]. Among NCDs, cardiovascular disease is one of the top 10 leading causes of death in Vietnam [5,12]. Access to health care services in Vietnam is at a low level [13]. In addition, the medication adherence rate, level of awareness, and treatment are relatively low in people with NCDs, and are significantly lower in rural settings [14,15,16].

Health literacy is an important component of public health practice, and it is needed to enable effective health promotional activities/programs and behavior changes [17,18,19,20,21,22]. Better HL is associated with better self-care, better health outcomes, and lower health care expenditure [21,23,24,25,26]. Health literacy is strongly associated with quality of life [27], morbidity, and mortality in rural patients [28]. However, HL level was found to be low in either developing or developed countries [29,30,31]. Vietnam was shown to have the lowest HL level among studied countries [29,32]. Moreover, HL was alarmingly low in people living in rural areas [33].

It is critical to assess HL and its associated factors that may provide helpful evidence when it comes to appropriate health resource allocation. This could improve the effectiveness of health promotion programs and further reduce health inequalities in the context of SDGs. However, a valid, comprehensive survey tool has not been available for use in rural settings. Our aim in this study was to validate the comprehensive short-form health literacy questionnaire (HLS-SF12) and examine the potential determinants of HL among people living in rural areas.

## 2. Methods

### 2.1. Study Design and Settings

A cross-sectional study was conducted in July 2019 at two community health stations at the Nham commune in the A Luoi district and the Thuong Long commune in the Nam Don district, both of which are located in the Thua Thien Hue province, Vietnam. These two communes are located in poor mountainous areas in the center of Vietnam, with limited resources, such as health stations, for providing adequate examinations and medication to residents.

### 2.2. Sampling and Sample Size

The sample size required for conducting the structural equation model (confirmatory factor analysis) was recommended to be 10 times the item number [34]. A sample of 120 participants was adequate for 12 items of the short-form health literacy questionnaire (HLS-SF12). However, our aim was to examine associated factors of health literacy. Therefore, the sample size was calculated using G-Power version 3.1 software (Heinrich-Heine-University, Düsseldorf, Germany) [35]. It was calculated that a sample size of 416 people was required, with a precision or effect size of 0.04, as suggested for a cross-sectional design [36], type I error of 5%, power of 80%, and with 10 predictors investigated in the current study.

Residents visiting community health stations were recruited during the medical tours of Hue University of Medicine and Pharmacy in July 2019. The people selected were those who resided in two mountainous communes in two districts in a central province in Vietnam, were aged 18 and above, did not have any emergency conditions or any mental health problems, and were able to listen and understand the local language or dialect. A total sample of 440 people was collected and analyzed.

### 2.3. Health Literacy Assessment

The short-form health literacy questionnaire (HLS-SF12), which has been validated in the general population of six Asian countries [32], was used to measure health literacy (HL). The values of Cronbach’s alpha and the goodness-of-fit index of the HLS-SF12 in the general Vietnamese population were 0.87 and 0.97, respectively [32]. People rated the perceived difficulty of each item on four-point Likert scales (1 = very difficult, 2 = difficult, 3 = easy, and 4 = very easy). The indices for HL were standardized to unified metrics from 0 to 50 using the formula; *Index* = (*mean* − 1) × (50/3), where *Index* is the specific index calculated, *mean* is the mean of all participating items for each individual, 1 is the minimal possible value of the mean (leading to a minimum value of the index of 0), 3 is the range of the mean, and 50 is the chosen maximum value of the new metric. Thus, an index value is obtained where 0 represents the lowest HL and 50 the highest HL [37]. The indices examined in the current study were general HL (GHL) and indices of three domains including health care HL (HC-HL), disease prevention HL (DP-HL), and health promotion HL (HP-HL).

### 2.4. Personal Characteristics

The following variables were also assessed; age, gender, ethnicity, marital status, education, occupation, the ability to pay for medication, social status, tendency to view health-related TV/radio, and community involvement.

### 2.5. Data Collection Procedure

The medical students who were utilized as interviewers were trained in data collection by a senior researcher. A four-hour training session took place on the university campus. A daily meeting was conducted in order to improve the quality of the data collected. The research team (researcher and medical students/interviewers) had a two-hour meeting with the medical tour team (volunteer doctors and nurses) and local volunteers regarding the surveys in order to organize the health check and interview sessions. The face to face interviews were conducted at the community health stations using printed questionnaires. Local volunteers helped with interpretation for participants who used the local dialect (about one fifth to one fourth of participants). A consent form was obtained by each participant, while adequate time (15–30 min) was allowed for answering all the questions.

### 2.6. Ethical Approval

The study was approved by the Institutional Ethical Review Committee of Hanoi School of Public Health, Vietnam (No. 379/2019/YTCC-HD3). All participants were asked for their permission and signed consent forms before their participation.

### 2.7. Statistical Analysis

Firstly, descriptive analysis was conducted to examine the distribution of study variables. The frequency and percentage, mean and standard deviation were reported. The independent-samples T-test and one-way ANOVA test were used to compare the distribution of health literacy between categories of participants’ characteristics, appropriately. Secondly, to evaluate the validity of the HLS-SF12, we used confirmatory factor analysis (CFA), with a maximum likelihood algorithm estimation to assess the construction of the questionnaire, focusing on three domains of health including health care, disease prevention, and health promotion [38]. The goodness-of-fit indices were reported, including: (i) Absolute model fit, root mean square error of approximation (RMSEA), and goodness-of-fit index (GFI); (ii) Incremental fit, adjusted goodness-of-fit index (AGFI), comparative fit index (CFI), incremental fit index (IFI), and normal fit index (NFI); and (iii) Parsimonious fit, or the chi-square goodness-of-fit test, and the chi-square/degrees of freedom ratio (χ^2^/df ratio). This method has also been used in a previous study [29]. In addition, the Pearson correlation coefficient was used to assess item-scale convergent validity [39]. Thirdly, the reliability of the HLS-SF12 was assessed using the internal consistency test (Cronbach’s alpha) [40], and the split-half reliability test [41,42]. Fourthly, we examined the floor and ceiling effects of the HLS-SF12 to reflect the responses of participants with the lowest and highest possible scores, respectively. A percentage of 15% or less at the floor or ceiling levels was recommended [43]. Finally, bivariate and multivariate linear regression models were used to explore the determinants of health literacy. The significance level was set at *p*-value < 0.05. Data were analyzed using the IBM SPSS Version 20.0 (IBM Corp, Armonk, NY, USA), and AMOS version 22.0 (IBM Corp, Armonk, NY, USA)[44].

## 3. Results

The average age of the study participants was 40.8 ± 13.4 years old; 43.6% were men, 85.9% were an ethnic minority, 87.7% were married, 19.1% were illiterate, 77.7% had participated in agroforestry work, 73% had difficulty paying for medication, 38.1% were perceived to have low social status, 40.2% never or rarely viewed health-related television or radio, and 52.7% were never or were rarely involved in community activities (Table 1). The overall HL index score was 24.2 ± 9.0. The distribution of HL differed among groups depending on age, gender, marital status, education, occupation, the ability to pay for medication, social status, those with a tendency to view health-related television/radio, and those involved with community activities (Table 1).

**Figure 1:**
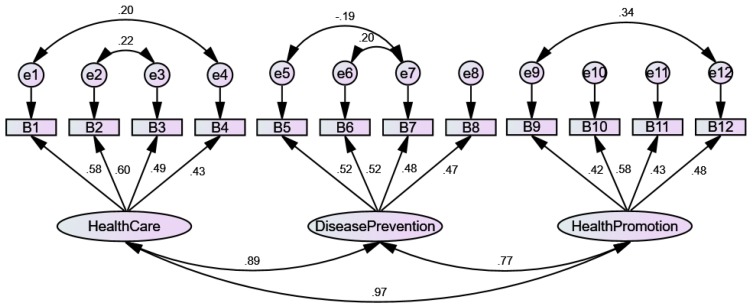
Structure equation model of the HLS-SF12 with 12 items loading into three domains of health (health care, disease prevention, health promotion). The questions from B1 to B12 of the HLS-SF12 are stated below. On a scale from very easy to very difficult, how easy would you say it is to: B1…find information on treatments of illnesses that concern you? B2…understand the leaflets that come with your medicine? B3…judge the advantages and disadvantages of different treatment options? B4…call an ambulance in an emergency? B5…find information on how to manage mental health problems like stress or depression? B6…understand why you need health screenings (such as breast exam, blood sugar test, blood pressure)? B7…judge which vaccinations you may need? B8…decide how you can protect yourself from illness based on advice from family and friends? B9…find out about activities (such as meditation, exercise, walking, Pilates, etc…) that are good for your mental well-being? B10…understand information in the media (such as Internet, newspaper, magazines) on how to get healthier? B11…judge which everyday behavior (such as drinking and eating habits, exercise, etc…) is related to your health? B12… join a sports club or exercise class if you want to?

The psychometric properties of the HLS-SF12 are presented in Table 2. Firstly, the construct validity was analyzed by CFA, and the results indicated a good model-data-fit [45]. The absolute model fit was satisfactory, with an RMSEA value of 0.09, and a GFI of 0.94. The incremental fit was adequate, with values of AGFI, CFI, IFI, and NFI ranging from 0.82 to 0.89. The parsimonious fit (χ^2^/df = 4.34) was also adequate (Table 2). The correlations among three HL domains of healthcare, disease prevention, and health promotion were significantly strong with values ranging from 0.71 to 0.96 (Figure 1).

Item-scale convergent validity was satisfactory, with the correlations of 12 items with overall HLS-SF12 scales ranging from 0.49–0.64. The item-scale correlations were stronger in three domains of healthcare (0.66–0.74), disease prevention (0.62–0.73), and health promotion (0.63–0.73), respectively (Table 2). The internal consistency reliability of the HLS-SF12 was adequate, with a Cronbach’s alpha value of 0.79. There was no floor or ceiling effect, as the percentages of people with the lowest scores or the highest scores of HL were 0.50% and 0.00%, which were less than 15% (Table 2).

Finally, the determinants of HL were examined using linear regression models. The results showed that HL was significantly lower in people who were married (regression coefficient, B = −3.12; 95% confidence interval (CI) = −5.69, −0.56, *p* = 0.017) as compared with those who had never married. In comparison with illiterate people, those with higher education attainment at Elementary School (B = 3.41; 95% CI = 1.33, 5.49; *p* = 0.001), Secondary School (B = 7.11; 95% CI = 5.07, 9.15; *p* < 0.001), High School (B = 7.64; 95% CI = 5.31, 9.97; *p* < 0.001), or Vocational/University (B = 10.44; 95% CI = 6.67, 14.22; *p* < 0.001) had higher HL scores. Compared with people with the ability to pay for medication at the very difficult level, those with the ability at the levels of fairly difficult (B = 4.17; 95% CI = 2.51, 5.83; *p* < 0.001), fairly easy (B = 5.64; 95% CI = 3.57, 7.72; *p* < 0.001), and very easy (B = 9.89; 95% CI = 6.97, 12.80; *p* < 0.001) had higher HL scores. Compared to people who had never viewed health-related TV/radio, those who viewed rarely (B = 5.23; 95% CI = 2.97, 7.49; *p* < 0.001), sometimes (B = 4.13; 95% CI = 2.10, 6.16; *p* < 0.001), and often (B= 6.15; 95% CI = 3.70, 8.60; *p* < 0.001) had higher HL scores (Table 3).

## 4. Discussion

The HLS-SF12 was shown to have satisfactory construct validity with a good model-data-fit [45]. In addition, all items correlated with an overall scale and had their own domain scales at moderate and high levels [46], which satisfied the criterion of item-scale convergent validity [39].

The internal consistency of the HLS-SF12 was at an adequate level (Cronbach’s alpha = 0.79) in the current study population, which was slightly lower than previous studies conducted in Europe (Cronbach’s alpha = 0.87 to 0.97) [37] and Asia (Cronbach’s alpha = 0.79 to 0.96) [29,32,47]. In addition, the reliability of the HLS-SF12 was strengthened by the minimal floor/ceiling effect [43].

In the current study, age was negatively associated with HL in the unadjusted model. However, the association was attenuated in the adjusted model. This indicates that age might not be a predictor of HL in rural areas, even though age was one of the important factors of HL in populations in Austria, Bulgaria, Greece, Poland, and Spain, and was partially important in the Netherlands [30,37,48], the USA [49,50], Canada [51], and Taiwan [19,47].

Education attainment and the ability to pay for medication were both highly associated with HL in both the current study and in previous studies in Europe [30,48] and Asia [29,47,52]. In addition, the tendency to view health-related television/radio was positively associated with health literacy, as it was also found to be in a previous study [19]. In the current study, people who were married had lower HL compared with those who had never married. However, marital status has not been significantly associated with HL in previous studies [47,53]. This indicates that in order to improve people’s HL in rural areas, particularly for those who are married, interventions to improve the economic, educational, and health-promoting mass media are highly important.

Health literacy was not significantly different between ethnic minority groups in the current study. This was also found in a previous study in the Netherlands [54]. In addition, HL was also not significantly different between groups of occupation and social status. On one hand, this might indicate that among social demographic factors, ethnic minority is not an important factor. On the other hand, this partly reflect the fact that people living in the same geographical area with the same infrastructure of healthcare systems likely receive the same information and in turn achieve the same level of HL [4]. Finally, community involvement was found to be a predicator of HL in the general population in Taiwan [19,47], but it was not found to be a predicator in people living in mountain areas in the current study. This suggests that health-promoting activities need to be integrated into community activities in rural areas.

The current study has some limitations. Firstly, the study was conducted during the medical tour that provided free health checks and medication for people who live in rural areas. Therefore, the sample collected might not be representative for the whole population, and the external validity of the survey tool may be limited. Secondly, the findings of the current study were based on a sample of 440 people who resided in two mountainous communities in Vietnam. Therefore, the association between personal characteristics and health literacy may not be generalizable to all rural areas in Vietnam. In addition, the associations found in a cross-sectional study can only raise the phenomenon or hypothesis for future study; the causality cannot be generated. Future study with a larger study population in rural areas is required to explore the health literacy levels and the associated factors that might contribute to potential interventions in order to improve the quality of healthcare services and people’s health in rural areas in Vietnam and across the Globe and to achieve SDGs by the year 2030 [2,55].

## 5. Conclusions

The HLS-SF12 was shown to be valid as a comprehensive survey tool to measure health literacy in a rural setting. Marital status, education, the ability to pay for medication, and the tendency to view health-related television/radio were all determinants of health literacy. The findings may be helpful for effective public health interventions to enhance people’s HL and reduce health inequalities in rural areas.

## Figures and Tables

**Table 1:** Characteristics and health literacy index score of participants.

	Total (*N* = 440)	GHL Index	
	Frequency (%)	Mean ± SD	*p*-Value ^1^
Age			0.007
18–39	223 (50.7)	25.0 ± 9.0	
40–59	179 (40.7)	24.1 ± 8.6	
≥60	38 (8.6)	20.1 ± 10.1	
Gender			<0.001
Women	248 (56.4)	22.5 ± 9.3	
Men	192 (43.6)	26.4 ± 8.2	
Ethnicity attainment			0.376
Kinh (Vietnamese)	62 (14.1)	23.3 ± 9.5	
Ethnic minority	378 (85.9)	24.4 ± 8.9	
Marital status			<0.001
Never married	54 (12.3)	31.7 ± 8.2	
Married	386 (87.7)	23.2 ± 8.6	
Education			0.004
Illiterate	84 (19.1)	17.2 ± 8.8	
Elementary School	102 (23.2)	20.9 ± 8.7	
Secondary School	128 (29.1)	26.1 ± 7.1	
High School	102 (23.2)	29.0 ± 7.1	
Vocational/University	24 (5.5)	32.6 ± 5.4	
Occupation			0.002
Agroforestry	342 (77.7)	23.5 ± 8.8	
Others (Officers/Small trade/Craft/Housework)	98 (22.3)	26.7 ± 9.3	
Ability to pay for medication			<0.001
Very difficult	143 (32.5)	19.2 ± 8.4	
Fairly difficult	178 (40.5)	24.8 ± 8.2	
Fairly easy	87 (19.8)	28.9 ± 7.3	
Very easy	32 (7.3)	30.7 ± 8.8	
Social status			<0.001
Low	167 (38.1)	21.5 ± 8.9	
Middle or High	271 (61.9)	26.0 ± 8.7	
Tendency to view health-related TV/radio			<0.001
Never	67 (15.2)	19.1 ± 9.8	
Rarely	110 (25.0)	25.3 ± 7.3	
Sometimes	192 (43.6)	24.9 ± 9.0	
Often	71 (16.1)	25.7 ± 9.3	
Community involvement			0.003
Never	148 (33.6)	23.2 ± 9.0	
Rarely	84 (19.1)	22.3 ± 9.8	
Sometimes	157 (35.7)	25.2 ± 8.1	
Often	51 (11.6)	27.4 ± 9.6	

Abbreviations: GHL, general health literacy; SD, standard deviation; TV, television. ^1^ *p*-values were calculated to compare the distribution of the general health literacy index between different categories of participants’ characteristics using the independent-samples T-test or One-way ANOVA test, appropriately.

**Table 2:** Goodness-of-fit indices, item-scale convergent validity, internal consistency reliability, and floor/ceiling effects of the HLS-SF12.

	Total Sample (*N* = 440)
Absolute model fit ^1^	
RMSEA	0.09
GFI	0.94
Incremental fit ^1^	
AGFI	0.89
CFI	0.85
IFI	0.86
NFI	0.82
Parsimonious fit ^1^	
χ^2^/df	4.34
Item-scale convergent validity, range of correlations (rho)	
GHL	0.49–0.64
HC-HL	0.66–0.74
DP-HL	0.62–0.73
HP-HL	0.63–0.73
Reliability, Cronbach’s alpha	
GHL	0.79
HC-HL	0.65
DP-HL	0.56
HP-HL	0.60
Floor effects, %	
GHL	0.50
HC-HL	7.70
DP-HL	1.40
HP-HL	1.80
Ceiling effect, %	
GHL	0.00
HC-HL	1.40
DP-HL	1.40
HP-HL	5.00

^1^ Structure Equation Model of the HLS-SF12 with 12 items loading into three domains of health (health care, disease prevention, health promotion). Abbreviations: HLS-SF12, short-form health literacy questionnaire; RMSEA, root mean square error of approximation; GFI, goodness-of-fit index; AGFI, adjusted goodness-of-fit index; CFI, comparative fit index; IFI, incremental fit index; NFI, normal fit index; χ^2^/df, relative chi-square; GHL, general health literacy; HC-HL, health care health literacy; DP-HL, disease prevention health literacy; HP-HL, health promotion health literacy.

**Table 3:** Determinants of health literacy of people living in rural areas (*N* = 440).

	Bivariate Model		Multivariate Model	
	B (95% CI)	*p-*Value	B (95% CI)	*p-*Value
Age				
18–39	Reference		Reference	
40−59	−0.92 (−2.68, 0.84)	0.306	0.82 (−0.61, 2.25)	0.259
≥60	−4.98 (−8.06, −1.89)	0.002	−2.31 (−4.89, 0.28)	0.080
Gender				
Women	Reference		Reference	
Men	3.90 (2.24, 5.57)	<0.001	0.14 (−1.40, 1.67)	0.860
Ethnicity				
Kinh (Vietnamese)	Reference		Reference	
Ethnic minority	1.10 (−1.33, 3.53)	0.376	1.43 (−0.51, 3.37)	0.148
Marital status				
Never married	Reference		Reference	
Married	−8.52 (−10.97, −6.07)	<0.001	−3.12 (−5.69, −0.56)	0.017
Education attainment				
Illiterate	Reference		Reference	
Elementary School	3.73 (1.49, 5.97)	0.001	3.41 (1.33, 5.49)	0.001
Secondary School	8.89 (6.76, 11.03)	<0.001	7.11 (5.07, 9.15)	<0.001
High School	11.79 (9.55, 14.03)	<0.001	7.64 (5.31, 9.97)	<0.001
Vocational/University	15.43 (11.91, 18.95)	<0.001	10.44 (6.67, 14.22)	<0.001
Occupation				
Agroforestry	Reference		Reference	
Others (Officers/Small trade/Craft/Housework)	3.13 (1.12, 5.14)	0.002	−1.21 (−3.10, 0.68)	0.209
Ability to pay for medication				
Very difficult	Reference		Reference	
Fairly difficult	5.55 (3.75, 7.34)	<0.001	4.17 (2.51, 5.83)	<0.001
Fairly easy	9.70 (7.53, 11.88)	<0.001	5.64 (3.57, 7.72)	<0.001
Very easy	11.49 (8.36, 14.62)	<0.001	9.89 (6.97, 12.80)	<0.001
Social status				
Low	Reference		Reference	
Middle or High	4.53 (2.84, 6.22)	<0.001	1.37 (−0.13, 2.87)	0.073
Tendency to view health-related TV/radio				
Never	Reference		Reference	
Rarely	6.19 (3.52, 8.87)	<0.001	5.23 (2.97, 7.49)	<0.001
Sometimes	5.75 (3.30, 8.20)	<0.001	4.13 (2.10, 6.16)	<0.001
Often	6.63 (3.69, 9.57)	<0.001	6.15 (3.70, 8.60)	<0.001
Community involvement				
Never	Reference		Reference	
Rarely	−0.91 (−3.30, 1.48)	0.454	−0.43 (−2.38, 1.51)	0.662
Sometimes	1.99 (−0.02, 3.99)	0.052	−0.63 (−2.31, 1.05)	0.460
Often	4.21 (1.36, 7.05)	0.004	2.05 (−0.43, 4.53)	0.105

Abbreviations: B, regression coefficient; CI, confidence interval; TV, television.
